# Impact of birthweight on health-care utilization during early childhood – a birth cohort study

**DOI:** 10.1186/s12887-019-1424-8

**Published:** 2019-03-01

**Authors:** Mario Rüdiger, Luise Heinrich, Katrin Arnold, Diana Druschke, Jörg Reichert, Jochen Schmitt

**Affiliations:** 1 Department for Neonatology and Pediatric Intensive Care, University Hospital Carl Gustav Carus, Technische Universität Dresden, Fetscherstr. 74, 01307 Dresden, Germany; 2 Center for Evidence-based Healthcare, University Hospital and Medical Faculty Carl Gustav Carus, Technische Universität Dresden, Fetscherstr. 74, 01307 Dresden, Germany

**Keywords:** Birthweight, Preterm birth, Epidemiology, Cost, Burden of disease, Birth cohort, Health care

## Abstract

**Background:**

Comprehensive data are needed to evaluate the burden of low birthweight. Analysis of routine data on health-care utilization during early childhood were used to test the hypothesis that infants with low birthweight have (i) increased inpatient health-care utilization, (ii) higher hospital costs and (iii) different morbidity pattern in early childhood when compared with normal birthweight infants.

**Methods:**

Children born between 2007 and 2013 that were insured at birth with the statutory health insurance AOK PLUS were included (*N* = 118,166, equaling 49% of the Saxon newborns) and classified into very low (< 1500 g, VLBW), low (1500-2499 g, LBW) birthweight and reference group (> 2500 g). Outcomes were: inpatient health-care utilization quantified by number and length of hospital stays; costs of hospitalizations including medication; reasons of hospitalizations for each year of life (YOL).

**Results:**

72, 38 and 22% of VLBW-, LBW- and reference group were hospitalized after perinatal period within the first YOL with a more than 5-fold increased risk in VLBW to be hospitalized for hemangioma, convulsions, hydrocephalus, hernia and respiratory problems. Median (IQR) cumulative cost of inpatient care during the first four YOLs was 2953 (1213-7885), 1331 (0–3451) and 0 (0–2062) Euro for respective groups. Inpatient early childhood health-care utilization (after first YOL) was higher in VLBW compared to healthy, normal birth weight infants (RR 3.92 [95%-CI 3.63, 4.23]), residents of rural areas (RR 1.37 [95%-CI 1.35, 1.40]) and in boys (RR 1.31 [95%-CI 1.29, 1.33]).

**Conclusion:**

This large population-based birth-cohort study indicates a high clinical and economic burden of low birthweight which is not restricted to the first year of life.

**Electronic supplementary material:**

The online version of this article (10.1186/s12887-019-1424-8) contains supplementary material, which is available to authorized users.

## Background

Low birthweight, most frequently resulting from preterm delivery and/or intra-uterine growth retardation, represents an important public health issue since it is associated with profound short term and long term consequences – not only for the affected child and the family, but also for society and health-care systems [1–3].

Despite the risks associated with low birthweight there is only limited evidence regarding its long-term impact on health-care utilization and associated costs. Most of the relevant data originates either from the past century [4, 5] or does not contain any information regarding the reasons for health-care utilization. A recent study by Barradas and coworkers [6] compared hospital utilization and costs associated with low birthweight between Medicaid and commercial insurance in USA, but data are restricted to the first month after birth. Klitkou et al. [7] have recently presented data on the use of hospital-based health services from a population-based cohort of very preterm infants; however, the data were compared with the general population based on official statistics in Norway.

Population-based studies on health care utilization that compare low birthweight children with normal birthweight children in a realistic setting and follow these children through the whole health-care system from birth for several years are desirable to inform the development and implementation of targeted preventive care models.

Health insurance data provide valuable information not only regarding the frequency but also regarding the reason for hospitalization and its associated costs. This offers the great opportunity of monitoring health-care utilization in a defined region for a well described population over time. Health-care insurance data are not affected by recall bias or social desirability bias, making subsequent analyses and conclusions very reliable and generalizable [8–11].

The Early comprehensive Care of Preterm Infants (EcoCare-PIn) study investigates the effects of low birthweight on quality of life, childhood development, and health-care utilization using secondary data from the major health insurance in the Free State of Saxony and combines this data with primary data from parental questionnaires [12].

The present analysis tests the hypothesis that infants with low birthweight have (i) increased inpatient health-care utilization, (ii) higher inpatient costs and (iii) a different morbidity pattern, thus leading to higher hospitalization rates in early childhood when compared with normal birthweight infants.

## Methods

### Study design and data source

The publicly funded cohort study EcoCare-PIn has been registered (Deutsches Netzwerk Versorgungsforschung: VfD_EcoCare-PIN_13_003463) and described elsewhere [12]. The study was approved by the responsible ethics committee (EK 67022014) and the Saxon Data Protection Commissioner (2–7410-74/1). The study was performed in accordance with the declaration of Helsinki [13].

The study cohort is based on health insurance data from the Free State of Saxony in Germany. Pseudonymized data-sets were provided by the German statutory health insurance AOK PLUS for all insured children within the Federal State of Saxony who were born between January 1st, 2007 and December 31st, 2013 as follows: birthweight, age, sex, first three digits of postal code, information on in- and out-patient medical care including admission and discharge dates for each hospital episode, inpatient diagnoses, inpatient health-care costs, as well as all prescriptions, outpatient diagnoses, specialties of outpatient physicians and health-care utilization dates. All children were followed until end of 2013, insurance expiry or death.

### Case definitions and study collectives

Infants were stratified according to birthweight into three birthweight groups:

- very low birthweight (VLBW), i.e. birthweight below 1500 g,
- low birthweight (LBW), i.e. birthweight 1500 to 2499 g,
- Reference group with a birthweight ≥2500 g

Health insurance data of the infants did not contain adequate information regarding gestational age, thus grouping was based solely on birthweight.

To study the association of low birthweight and inpatient care after perinatal hospitalization by year of life (YOL), children had to be insured at their birth and needed to be continuously insured during this YOL or until their death within this YOL, respectively. The day of admission was used to allocate hospitalizations to YOL.

### Primary outcome measures

*Length of stay (LOS)* by YOL was calculated by summing-up the number of inpatient days of each children’s successive admission within the respective YOL. *Costs of hospitalization* are presented as the amount the insurance company pays the hospital (based on the system of diagnosis-related groups (DRG)) to cover all inpatient costs including salary of health care professionals, medications and other treatment costs in Euros for the respective YOL. *Cumulative LOS and costs* were calculated over the first four YOLs. To determine the *hospital morbidity pattern,* the main diagnosis of each hospital stay according to ICD-10-GM (International Classification of Diseases, 10th revision, German Modification) was considered. Data protection requirements restricted us to provide exact numbers of children if the number in the specific ICD block was below ten.

### Statistical analysis

Boxplots and bar charts were used to illustrate frequencies, lengths and costs of hospitalizations stratified by exposure group.

To analyse the association of birthweight and inpatient care during the first YOLs, all children were followed from their first birthday on as long as possible, resulting in different numbers of analysed infants per YOL (for details see Additional file 1: Table S1). A negative binomial regression was used to model the cumulative number of days spent in hospital during observation time. The natural logarithm of the observation time was included as a covariate into the model, since intensity of events varied proportionally with time. In addition, the presumed confounding factors were considered: sex, area of living, the presence of previous perinatal hospitalization. To categorize children’s residence into urban and rural districts, ZIP-Codes were used. As there was significant interaction of variables “birthweight” and “perinatal hospitalization”, both variables were included combined into the model. We performed Poisson as well as negative-binomial regression analysis and selected models by the help of Akaike Information Criterion and Bayesian Information Criterion. The more complex zero-inflated regression model did not provide any crucial advantage over our chosen negative binomial regression model.

Unadjusted Risk Ratios were calculated to compare the risk of being hospitalized due to the respective disease groups within the distinct YOLs among the three birthweight groups. We used Bonferroni correction to account for the high number of RR (based on the number of comparisons within the respective YOL).

All analyses were conducted using Stata, version 14. A two-sided *p* value of less than 0.05 was considered significant.

## Results

### Study population

The total study cohort consisted of 118,166 infants including 1265 (1.1%) and 6341 (5.4%) children with VLBW and LBW, respectively. The study population represented 49% of all infants born in Saxony during the observation period. Source population included similar relative percentages of VLBW (1.0%) and LBW-children (5.2%), thus suggesting representativeness of the study population.

Data from 116,269 infants were used for analyses of *perinatal hospitalization* (further details regarding patient exclusion are found in supplementary material Additional file 2: Perinatal Hospitalization). Overall, 20% of infants (19% of all male and 22% of all female infants) were hospitalized in the perinatal period (excluding normal well-baby care). Perinatal hospitalization rates considerably differed between birthweight-groups with 100% of VLBW-infants, 79% of LBW-infants and 16% of the infants in the reference group being hospitalized. Total frequency of *perinatal hospitalization* decreased over time from 23% in 2007 to 19% in 2013, mainly due to a reduction of in-patient treatment in the reference group (details are found in Additional file 3: Table S2).

Whereas the majority of LBW- (96%) and reference infants (94%) was treated in only one hospital, 17% of VLBW infants were transferred at least to one and almost 4% to two or more other hospitals during *perinatal hospitalization*.

**Fig. 1:**
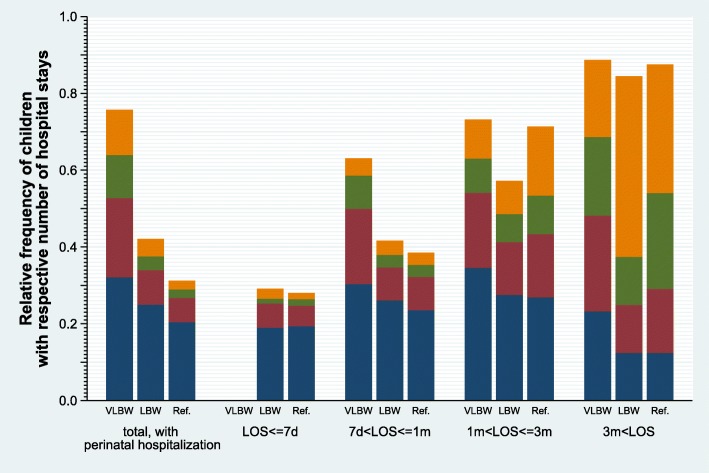
Number of hospital stays during 1st year following perinatal hospitalization by length of perinatal hospitalization and birthweight. Shown are the relative percentages of perinatally hospitalized VLBW-(*n* = 892), LBW-(*n* = 3891) and reference-infants (*n* = 14,501) with 1(blue), 2(red), 3(green) or more than 3(orange) hospitalizations during the first year after perinatal hospitalization (VLBW: χ^2^ = 55, LBW: χ^2^ = 286, NBW: χ^2^ = 777, all *p*-values < 0.001)

One hundred ninety nine infants (0.9%) died during *perinatal hospitalization*. Most of these (*n* = 118; 59%) were VLBW-infants; resulting in an in-hospital mortality rate of 9.8% in the VLBW group (detailed information is given in Additional file 4: Table S3). Thus, a total of 23,208 infants were used for subsequent analyses on perinatal hospitalization outcomes. Perinatal length of stay (LOS) substantially differed between groups with longer hospitalization in infants with lower birthweight (see Additional file 5: Figure S1 A and B). In-patient treatment costs during *perinatal hospitalization* increased with decreasing birthweight (see Additional file 5: Figure S1 A and B) with a trend of increase over the years of the study (see Additional file 6: Figure S2). Length of perinatal hospitalization was significantly (χ^2^ -test: all *p*-values < 0.001) associated with the number of hospitalizations in the subsequent one-year period in all three birthweight groups (Fig. 1).

### Frequency, length and cost of hospitalization after the perinatal period

**Fig. 2:**
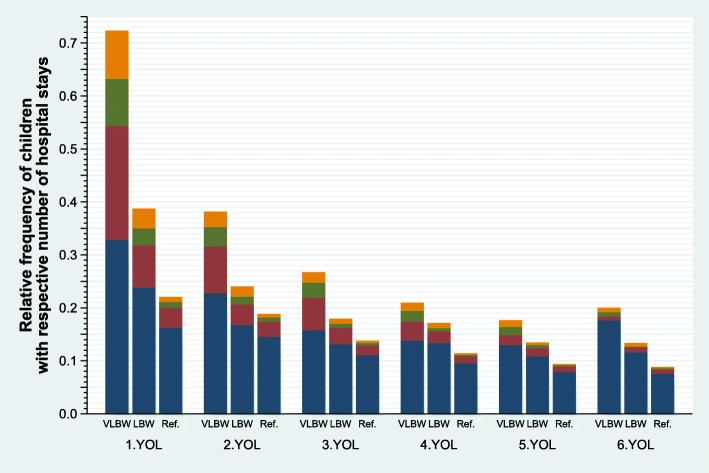
Number of hospital stays excluding perinatal hospitalization by YOL and birthweight. Shown are the relative percentages of VLBW-, LBW- and reference-infants with 1(blue), 2(red), 3(green) or more than 3(orange) hospitalizations in the respective year of life (YOL) excluding perinatal hospitalization (number of infants analysed per YOL are shown in supplement)

Inpatient health-care utilization of VLBW- and LBW-children was higher throughout the first 6 YOLs when compared to the reference group. Almost 3 out of 4 (72%) VLBW-infants were hospitalized again after the perinatal period within the first YOL; this rate was much lower in the LBW- (39%) and reference group (22%). In subsequent YOLs, frequency of hospital treatment decreased in all three groups; however, VLBW and LBW infants continued to require hospital treatment more often (Fig. 2).

Neither the LOS, nor the associated health-care costs of each individual hospitalisation showed relevant differences between birthweight groups (Tab. 1). Due to the higher number of hospitalizations, infants with lower birthweight had higher total costs for hospital treatment in each YOL (Tab. 1).

**Table 1:** A/B: Median length of hospital stay in days (A) and median inpatient health care costs in Euro (B) by YOL for infants with at least one hospital treatment in the respective YOL

YOL	VLBW			LBW			Reference Group		
	N (%)	Individual treatment	Complete YOL	N (%)	Individual treatment	Complete YOL	N (%)	Individual treatment	Complete YOL
(A)									
1	666 (72.3)	3 (2-5)	5 (3-10)	1,951 (38.7)	3 (2-7)	4 (2-10)	19, 688 (22.0)	3 (2-6)	4 (2-7)
2	283 (38.1)	3 (2-6)	5 (2-9)	964 (24.0)	3 (2-6)	4 (2-8)	13,357 (18.8)	3 (2-5)	3 (2-6)
3	157 (26.7)	3.5 (2-6)	5 (2-9)	544 (17.9)	3 (2-5)	3 (2-7)	7,493 (13.7)	2 (2-4)	3 (2-5)
4	86 (20.9)	3 (2-5)	5 (2-7)	375 (17.1)	3 (2-5)	3 (2-5)	4,487 (11.4)	2 (1-4)	3 (2-5)
5	46 (17.6)	3 (2-6)	4 (2-8)	192 (13.4)	2 (1-5)	3 (2-6)	2,397 (9.4)	2 (1-4)	3 (2-5)
6	26 (20.0)	3 (1-4)	3 (1-4)	94 (13.3)	3 (2-5)	3 (2-6)	1,086 (8.8)	2 (1-4)	3 (2-5)
(B)									
1	666 (72.3)	1,565 (1,197-2,243)	2,696 (1,539-4,851)	1,951 (38.7)	1,721 (1,259-2,314)	2,236 (1,506-4,203)	19, 688 (22.0)	1,716 (1,224-2,244)	1,941 (1,404-3,264)
2	283 (38.1)	1,713 (1,295-2,327)	2,407 (1,493-4,214)	964 (24.0)	1,677 (1,344-2,186)	1,873 (1,428-3,443)	13,357 (18.8)	1,612 (1,271-2,023)	1,763 (1,356-2,649)
3	157 (26.7)	1,826 (1,367-2,380)	2,420 (1,502-4,472)	544 (17.9)	1,689 (1,268-2,244)	1,842 (1,307-3,059)	7,493 (13.7)	1,596 (1,223-2,074)	1,724 (1,252-2,477)
4	86 (20.9)	1,917 (1,294-2,470)	2,262 (1,373-4,421)	375 (17.1)	1,516 (1,241-2,208)	1,762 (1,283-2,480)	4,487 (11.4)	1,480 (1,220-2,148)	1,618 (1,248-2,356)
5	46 (17.6)	1,826 (1,346-2,433)	2,176 (1,416-3,981)	192 (13.4)	1,450 (1,192-2,253)	1,678 (1,274-2,516)	2,397 (9.4)	1,545 (1,227-2,190)	1,688 (1,253-2,391)
6	26 (20.0)	1,643 (1,220-2,312)	1,853 (1,236-2,485)	94 (13.3)	1,883 (1,328-2,348)	1,869 (1,351-2,474)	1,086 (8.8)	1,583 (1,230-2,201)	1,715 (1,260-2,386)

Shown are numbers (N) and relative percentage (%) of infants with at least one hospital treatment in the respective year of life (YOL), excluding perinatal hospitalization. For these hospitalized children the length of stay in days (A) and the health-care costs in Euro (B) are given as median (interquartile range) for each individual hospitalisation and the complete YOL

Cumulative length of stay and cost of inpatient care during the first four YOLs differed significantly between birthweight groups (Tab. 2); median LOS was 6 days and cost approximately 3000 Euro higher in VLBW than in the reference group.

**Table 2:** Cumulative length and cost of hospital treatment for the first four years of life

	All infants			At least one hospitalization after perinatal period		
	n_total_	Cumulative LOS [days]	Cumulative Cost [Euro]	n_hospitalized_ (%)	Cumulative LOS [days]	Cumulative Cost [Euro]
VLBW	411	6 (2–17)	2953 (1213 -7885)	346 (84.2%)	8 (3–20)	3934 (1984-9187)
LBW	2192	2 (0–8)	1331(0–3451)	1307 (59.6%)	6 (3–13)	2842 (1655-5888)
Reference	39,210	0 (0–4)	0 (0–2062)	18,023 (46.0%)	4 (2–9)	2265 (1482-4079)
*p*-Value	–	< 0.001	< 0.001	–	< 0.001	< 0.001

Shown are numbers (n_total_) of all analysed infants and number (n_hospitalized_) and relative percentage (%) of infants with at least one hospital treatment in the first four year of life (YOL), excluding perinatal hospitalization. For these children the cumulative length of stay and health-care costs are given as median (interquartile range). We used Kruskal-Wallis-test of independence to compare the costs and LOS over the first four years of the exposure groups

The health insurance company spent approximately 3.3, 10.9 and 85.8% of the entire birth-cohort budget (equaling 84.5 Million Euro) for the VLBW-, LBW- and reference group (representing 1.0% (*n* = 411), 5.2 (*n* = 2192) and 93.8% (*n* = 39,210) of the study population) respectively for their first four YOLs after *perinatal hospitalization*.

Regression analyses (Tab. 3) indicated significantly higher inpatient health-care utilization during early childhood (after first YOL) in children with lower birthweight, children living in rural areas and in boys. LBW-children without perinatal hospitalization had significantly less inpatient health-care utilization than children with normal birth weight who did not require a perinatal hospital treatment.

**Table 3:** Results of the regression analysis for the cumulative number of days spent in hospital in early childhood

	RR [95% CI]
Natural logarithm of observation time measured in continuous years	0.78 [0.77, 0.79]
Weight group * perinatal hospitalization (reference: Reference group without perinatal hospitalization)	
VLBW with perinatal hospitalization	3.92 [3.63, 4.23]
LBW without perinatal hospitalization	1.31 [1.21, 1.42]
LBW with perinatal hospitalization	2.34 [2.25, 2.44]
Reference group with perinatal hospitalization	1.49 [1.46, 1.53]
Sex (reference: female)	1.31 [1.29, 1.33]
Area (reference: urban)	1.37 [1.35, 1.40]

Shown are the results of the negative-binomial regression (*n* = 84,343), all *p*-values < 0.001

### Hospital morbidity pattern

In the first YOL, the most prominent reason for hospitalization of VLBW-infants (after perinatal hospitalization) was vaccination (Z20-Z29). Out of 666 VLBW-infants with at least one hospitalization in the first YOL, 116 infants (17%) were hospitalized just for the reason of vaccination or further circumstances not encoded as disease (Z-codes of ICD-10-GM). The remaining 550 infants had at least one hospitalization for other, i.e., morbidity related reasons.

In the first YOL, VLBW-infants had a more than 5-fold increased risk (compared to reference group) to be hospitalized for the following reasons: benign neoplasms (mainly hemangioma), episodic and paroxysmal disorders (mainly sleep disorders), other disorders of the nervous system (mainly hydrocephalus), problems originating from prematurity, hernia and symptoms and signs involving the circulatory and respiratory systems. Interestingly, the frequency of VLBW-infants treated in hospital for injuries of the head tended to be lower than in reference group (Tab. 4).

**Table 4:** Causes of hospitalization

			VLBW N [%]	LBW N [%]	Reference Group N [%]	RR^a^ VLBW vs. NBW	RR^a^ LBW vs. NBW
First YOL	Cardiorespiratory system	Acute upper respiratory infections (J00-J06)	43 [4.7]	142 [2.8]	1603 [1.8]	2.6 [1.58,4.28]	1.57 [1.18,2.09]
		Influenza and pneumonia (J09-J18)	50 [5.4]	138 [2.7]	1318 [1.5]	3.68 [2.32,5.84]	1.86 [1.39,2.48]
		Other acute lower respiratory infections (J20-J22)	115 [12.5]	310 [6.1]	2790 [3.1]	4 [2.98,5.36]	1.97 [1.63,2.38]
		Congenital malformations of the circulatory system (Q20-Q28)	10 [1.1]	38 [.8]	211 [.2]	4.6 [1.59,13.26]	3.19 [1.79,5.69]
		Symptoms and signs involving the circulatory and respiratory systems (R00-R09)	88 [9.6]	173 [3.4]	897 [1]	9.52 [6.7,13.52]	3.42 [2.61,4.47]
	Central nervous system	Episodic and paroxysmal disorders (G40-G47)	40 [4.3]	60 [1.2]	298 [.3]	13.02 [7.56,22.42]	3.57 [2.24,5.67]
		Other disorders of the nervous system (G90-G99)	14 [1.5]	15 [.3]	68 [.1]	19.97 [7.65,52.14]	3.91 [1.53,9.98]
	Gastrointestinal system	Intestinal infectious diseases (A00-A09)	60 [6.5]	334 [6.6]	3594 [4]	1.62 [1.07,2.45]	1.65 [1.37,1.98]
		Hernia (K40-K46)	102 [11.1]	197 [3.9]	698 [.8]	14.18 [10.18,19.75]	5 [3.85,6.49]
		Symptoms and signs involving the digestive system and abdomen (R10-R19)	15 [1.6]	69 [1.4]	551 [.6]	2.64 [1.12,6.21]	2.22 [1.46,3.37]
	Prematurity-related problems	Respiratory and cardiovascular disorders specific to the perinatal period (P20-P29)	102 [11.1]	147 [2.9]	286 [.3]	34.6 [24.06,49.77]	9.11 [6.54,12.67]
		Other disorders originating in the perinatal period (P90-P96)	11 [1.2]	48 [1]	467 [.5]	2.29 [.84,6.2]	1.82 [1.11,2.99]
	Others	Benign neoplasms (D10-D36)	17 [1.8]	46 [.9]	252 [.3]	6.54 [2.89,14.82]	3.23 [1.91,5.47]
		General symptoms and signs (R50-R69)	38 [4.1]	135 [2.7]	1034 [1.2]	3.57 [2.09,6.07]	2.31 [1.72,3.11]
		Injuries to the head (S00-S09)	17 [1.8]	146 [2.9]	2774 [3.1]	.59 [.27,1.31]	.93 [.71,1.23]
		Persons with potential health hazards related to communicable diseases (Z20-Z29)^b^	183 [19.9]	64 [1.3]	39 [0]	455.24 [257.45,805]	29.08 [14.93,56.64]
Second YOL	Cardiorespiratory system	Acute upper respiratory infections (J00-J06)	30 [4]	138 [3.4]	1768 [2.5]	1.62 [.93,2.83]	1.38 [1.05,1.8]
		Influenza and pneumonia (J09-J18)	32 [4.3]	122 [3]	1429 [2]	2.14 [1.25,3.68]	1.51 [1.13,2.01]
		Other acute lower respiratory infections (J20-J22)	66 [8.9]	156 [3.9]	1488 [2.1]	4.24 [2.93,6.15]	1.85 [1.43,2.39]
	Central nervous system	Episodic and paroxysmal disorders (G40-G47)	11 [1.5]	29 [.7]	192 [.3]	5.48 [2.12,14.19]	2.67 [1.44,4.93]
	Gastrointestinal system	Intestinal infectious diseases (A00-A09)	50 [6.7]	218 [5.4]	2983 [4.2]	1.6 [1.05,2.45]	1.29 [1.05,1.59]
	Others	Diseases of middle ear and mastoid (H65-H75)	10 [1.3]	32 [.8]	469 [.7]	2.04 [.76,5.44]	1.21 [.69,2.11]
		Congenital malformations of genital organs (Q50-Q56)	24 [3.2]	32 [.8]	269 [.4]	8.54 [4.46,16.32]	2.1 [1.18,3.74]
		General symptoms and signs (R50-R69)	14 [1.9]	45 [1.1]	527 [.7]	2.54 [1.11,5.82]	1.51 [.94,2.43]
		Injuries to the head (S00-S09)	23 [3.1]	106 [2.6]	1877 [2.6]	1.17 [.62,2.22]	1 [.74,1.35]
Third YOL	Cardiorespiratory system	Acute upper respiratory infections (J00-J06)	19 [3.2]	63 [2.1]	757 [1.4]	2.33 [1.18,4.59]	1.49 [1.02,2.19]
		Influenza and pneumonia (J09-J18)	34 [5.8]	70 [2.3]	724 [1.3]	4.36 [2.63,7.23]	1.73 [1.2,2.5]
		Other acute lower respiratory infections (J20-J22)	21 [3.6]	49 [1.6]	619 [1.1]	3.15 [1.65,6.01]	1.42 [.92,2.2]
		Other diseases of upper respiratory tract (J30-J39)	16 [2.7]	78 [2.6]	1271 [2.3]	1.17 [.56,2.44]	1.1 [.78,1.55]
	Gastrointestinal system	Intestinal infectious diseases (A00-A09)	23 [3.9]	82 [2.7]	1130 [2.1]	1.89 [1.02,3.49]	1.3 [.93,1.82]
	Others	Congenital malformations of genital organs (Q50-Q56)	16 [2.7]	21 [.7]	190 [.3]	7.82 [3.65,16.76]	1.98 [1,3.91]
		General symptoms and signs (R50-R69)	10 [1.7]	24 [.8]	223 [.4]	4.16 [1.61,10.78]	1.93 [1.02,3.64]
		Injuries to the head (S00-S09)	11 [1.9]	57 [1.9]	965 [1.8]	1.06 [.43,2.58]	1.06 [.71,1.58]
Fourth YOL	Cardiorespiratory system	Acute upper respiratory infections (J00-J06)	11 [2.7]	24 [1.1]	342 [.9]	3.08 [1.38,6.87]	1.26 [.72,2.2]
		Influenza and pneumonia (J09-J18)	19 [4.6]	39 [1.8]	308 [.8]	5.91 [3.2,10.91]	2.27 [1.45,3.56]
		Other diseases of upper respiratory tract (J30-J39)	13 [3.2]	77 [3.5]	1003 [2.5]	1.24 [.6,2.57]	1.38 [1.01,1.88]

^a^Bonferroni-correction of significance level (α = 0.05) due to distinct number of comparisons: 1.YOL α = 0.001, 2.YOL: α = 0.002, 3.YOL: α = 0.003, 4.YOL: α = 0.008

Shown are numbers [N] and relative percentage [%] of infants that had been hospitalized at least once due to the depicted ICD blocks within respective year of life. All ICD blocks with at least 10 children in each weight group were chosen; all blocks of chapter XXI of ICD-10-GM were excluded, except from block Z20-Z29, which contains vaccination(^b^). Risk ratios are shown with Bonferroni corrected confidence intervals. Note: Due to decreasing overall case numbers there are less ICD blocks with at least 10 children with increasing YOL

In subsequent YOLs respiratory tract infections represented the major reason for admission in all birthweight groups, with notably higher rates among VLBW- and LBW-children. In VLBW, an over 5-fold increased likelihood to be hospitalized for the following disease groups was seen as compared to reference group: neurological problems in the 2nd YOL, for congenital malformations of genital organs in the 2nd and 3rd YOL and influenza and pneumonia in the 4th YOL. Comparison of LBW-infants with reference group infants revealed a similar trend; however, the effect was less prominent (Tab. 4).

## Discussion

Low birthweight represents a well-known risk for subsequent health problems and urges for an appropriate framework of care to reduce long-term burden; not only for affected families, but also for society [1, 3]. To reduce that burden, priorities of care and research have to be identified, using data on health-care utilization of infants with low birthweight. This data should be (i) population-based, (ii) include data from infants with normal birthweight, (iii) reflect current standard of care and (iv) consider trans-sectoral care. The EcoCare-PIn study investigates effects of low birthweight on quality of life, childhood development, and health-care utilization using secondary data from the major health insurance in Saxony (AOK PLUS) and combines this data with primary data from parental questionnaire [12].

Here we investigated the hypothesis, that infants with low birthweight have (i) increased inpatient health-care utilization, (ii) higher hospital costs and (iii) a different morbidity pattern leading to hospitalization in early childhood when compared with normal birthweight infants. Our analysis revealed several important results. Firstly, children with very low birthweight had a 3.9 fold increased inpatient health-care utilization compared to healthy normal birthweight infants. Secondly, severity of each illness episode after perinatal hospitalisation seems to be not higher in VLBW-infants; since neither LOS nor subsequent health-care costs of each individual hospitalisation showed relevant differences between birthweight groups. However, due to higher number of hospitalizations, cumulative costs for hospital treatment in the first four YOLs of VLBW- and LBW-infants are about 3000 or 1300 Euro higher than in reference group infants, respectively. Thirdly, low birthweight is associated with a distinct hospital morbidity pattern in early childhood that differs from reference infants.

Furthermore, the risk to be hospitalized in early childhood depends not only on birthweight, but also on other factors such as sex and area of living (rural versus urban). Finally, perinatal hospitalization per se (regardless of birthweight) increases the risk of hospital treatment during early childhood.

### Implications of results

Changes in neonatal care aim to improve neonatal outcome, however, good data on long-term morbidity are difficult to obtain. The present study shows how routine data on health-care utilization can be used for a population-based description of the health status of children. Based on this evidence, targeted preventive care models can be developed, implemented and finally evaluated. Since our analysis uses a reference population for comparison, data are comparable with future studies from other regions.

To reduce hospital treatment in early childhood and its subsequent health-care costs, infants with low birthweight or perinatal hospitalization should have a special follow-up based on their distinct morbidity patterns. As already known, low birthweight increases the risk of neurological and respiratory problems [14]. However, when compared to reference group infants, VLBW infants also have an increased risk to be hospitalized for hernia and hemangioma within the first YOL, as well as for problems of the cardiovascular system. A similar pattern has been described for preterms in Norway, however Klitkou et al. provide no information for healthy infants [7]. Furthermore, vaccination represents a major reason for hospitalization within the first YOL in VLBW-infants in the present study. Based on the increased risk of postimmunisation apnea in preterm infants [15], hospital-based monitoring of cardiorespiratory function has been generally recommended in Germany for all extremely preterm infants for the first vaccination (or even during subsequent vaccinations if apnoea occurred during the first one). Our study provides important new evidence that physicians in Germany follow that recommendation. To better compare our results with data from countries without any observational admission after vaccination, an additional analysis was performed, excluding the observational admissions (for details see Additional file 7: Figure S3).

Our analysis moreover revealed significantly higher inpatient health-care utilization in rural areas when compared to urban areas. However, prior to drawing any conclusions, several probable explanations have to be discussed. The categorization of children’s residence into urban and rural areas based on the ZIP-code is a simplification. Differences in primary care givers (paediatrician vs. general practitioner) could explain the differences and thus, have to be tested. Higher health-care utilization costs were recently described in infants with mothers living in low socioeconomic neighborhood [2]. Therefore, the effect of parental socioeconomic status on health-care utilization will be analysed in a sub-population of the EcoCare-PIn cohort.

Whereas data from Norway did not show an impact of the distance between home and hospital on health-care utilization, the overall LOS in the first YOL was slightly higher (almost 8 days) than in Saxony [7].

### Study strengths

An inverse relationship between birthweight and subsequent health-care costs has already been described [4]. Nevertheless, the present study – which is based on a large cohort of infants in a geographically well-defined area and includes a comprehensive and validated record of data on health-care utilization and costs – has several benefits when compared to previous research. First, previous studies focused mainly on costs of perinatal hospitalization of preterm infants [16–18] or for the entire first year of life [6, 19, 20]. Our approach analyses perinatal and subsequent hospitalizations separately and therefore extends previous studies in terms of length of observation period. Second, grouping of infants is based on actual birthweight and does not depend on ICD coding and its well-known restrictions. Third, in contrast to previous publication [21, 22] our analysis uses current data, which is crucial for decisions on health policy. However, even in the short time period we noted some changes in cost over the years (see Additional file 6: Figure S2). Finally, our study does not only present health-care costs, but also morbidity patterns which allow development of targeted preventive care models.

### Limitations of the study

Beside major advantages, some methodological limitations have to be discussed. To fully understand the burden of low birthweight, data of ambulant treatment and primary data regarding the well-being of infants and family are needed. These data are included in the EcoCare-PIn-study; however, presentation would be beyond the scope of the current report.

Routine data are collected for billing and reimbursement, what could influence the data quality. In our study, analysis of hospital morbidity pattern is based on the major ICD-code of each hospitalization. Therefore, our approach may be subject to up-coding. In addition, it neglects other relevant side-codes. However, these effects will most likely be non-differential, i.e. not alter the results in general since all three weight groups will be affected.

Health insurance data did not contain any valid information regarding gestational age; therefore infants were grouped according to birthweight, even though gestational age is generally preferred to classify preterm birth. However, birthweight can be considered as an adequate proxy for preterm birth [23, 24]. Furthermore, no information can be provided regarding the percentage of infants being small for gestational age (SGA) which is associated with increased risk of adverse outcomes. Thus it cannot be excluded that some of the higher costs in low birthweight are due to SGA-infants, since Marzouk et al. have recently shown that “being small for gestational age is an independent contributor to 1-year hospital costs” [20].

Our study cohort is based on patients insured with one health care insurance company (AOK PLUS). The study cohort covers almost half of the infants born in Saxony; however, no data are available from the remaining half which is insured with about 10 other companies. The demographic characteristics regarding sex and birthweight of the children born alive and insured at the AOK PLUS are in accordance with the reference data from the Federal Statistical Office of Germany (see Additional file 8: Table S4). Since no major socio-economic or geographical factor is influencing the choice of statutory health insurance company, we consider our data generalizable at least for patients with statutory health insurance in Saxony. When compared with other federal states of Germany, it has to be taken into account that neonatal mortality is lowest in Saxony (1.34 per thousand live births vs. 2.31 for entire Germany in 2008–2012) [25] which may limit generalisability of our findings. However, the same clinical guidelines, quality assurance measures as well as reimbursement regulations apply throughout Germany. Therefore we believe that at least the patterns of hospitalization are most likely similar in other federal states of Germany. Whereas the present data differ from reports from USA, comparison with other industrialized countries in Europe reveals similar trends but direct comparison is rather difficult due to differences in analysing and presenting the data [7, 19, 20]. A more throughout analysis of health care expenditure and outcome of preterm infants in different countries would be of great interest for future studies.

Finally, data for the fifth and sixth YOL are rather limited, despite of a data base of more than 100,000 children; only data obtained between 2007 and 2013 were available for analysis in here. However, follow-up analysis of our cohort is planned, to have sufficient statistical power to study effects of low birthweight even in adolescents.

## Conclusion

This large population-based birth cohort study indicates high clinical and economic burden of low birthweight which is not restricted to the first year of life. Inpatient health-care utilization is 3.9 fold increased in VLBW-infants, leading to cumulative costs for hospital treatment in the first four YOL of VLBW- and LBW-infants that are about 3000 and 1300 Euro higher than in reference group infants. Whereas low birthweight is associated with a distinct hospital morbidity pattern in early childhood, severity of each illness episode seems to be not higher in VLBW-infants; since neither LOS nor subsequent health-care costs of each individual hospitalisation showed relevant differences between birthweight groups. Finally, the risk of being hospitalized in early childhood depends not only on birthweight, but also on other factors such as the sex, area of living and the need for perinatal hospitalization.

## Additional files

Additional file 1: **Table S1.** Number of children analysed by year of life: Numbers of children continuously insured during the YOL of interest or continuously insured until their death (*n* = 134) within this YOL are shown. *Children that didn’t survive perinatal hospitalization (if present) were excluded. (DOC 34 kb)

Additional file 2: Perinatal Hospitalization. Gives information regarding the method of analysing perinatal hospitalization. (DOC 31 kb)

Additional file 3: **Table S2.** Perinatal hospitalization: Shown are number of infants insured during their first week of life (N) and that were perinatally hospitalized (N with periH, % with periH) stratified by year of birth and exposure group. (DOC 67 kb)

Additional file 4: **Table S3.** Death during perinatal hospitalization (in-hospital mortality): Shown are number of infants that died within perinatal hospitalization. For these children the health care cost for perinatal hospital treatment and the length of stay are represented as median with interquartile range (IQR). (DOC 36 kb)

Additional file 5: **Figure S1.** A and B Length and costs of perinatal hospitalization and by birthweight: Shown are Boxplots of the length and costs of perinatal hospitalization. Children with missing record were excluded. (DOC 37 kb)

Additional file 6: **Figure S2.** Boxplots of the costs (in thousand Euro) of perinatal hospitalization by year of birth and birthweight: Children with missing record of birthweight were excluded. Outside values (observations below 1.Quartile − 1.5 IQR or above 3.Quartile + 1.5 IQR) are not shown in the graph. Simple unadjusted linear regression was calculated for these displayed costs and regression coefficients with 95% CI are reported. Note different scales for costs. (DOC 47 kb)

Additional file 7: **Figure S3.** Number of hospital stays excluding perinatal hospitalization and observational admissions by YOL and birthweight: Shown are the relative percentages of VLBW-, LBW- and reference-infants with 1(blue), 2(red), 3(green) or more than 3(orange) hospitalizations in the respective year of life (YOL) excluding perinatal hospitalization and excluding all hospitalizations just for the reason of vaccination or further circumstances not encoded as disease (Z-codes of ICD-10-GM). (DOC 34 kb) (PDF 114 kb)

Additional file 8: **Table S4.** Characteristics of the study population: Shown are the total numbers of children born alive in Saxony stratified by year of birth, sex and birthweight given by the Federal Statistical Office of Germany and the Statistical Office of the Free State of Saxony. The same numbers are given for the study population. (DOC 37 kb)

## Abbreviations

EcoCare-PIn: Early comprehensive Care of Preterm Infants
GM: German Modification
ICD: International Classification of Diseases
IQR: Inter Quartile Range
LBW: low birthweight
LOS: Length of stay
SGA: Small for gestational age
VLBW: very low birthweight
YOL: Year of life
