# Supplementary material for: Impact of birthweight on health-care utilization during early childhood – a birth cohort study
Source: BMC Pediatr. 2019 Mar 1;19:69. doi: 10.1186/s12887-019-1424-8 (PMC6397462; doi:10.1186/s12887-019-1424-8)
Supplement: Supplementary file 1 — Table S1. Number of children analysed by year of life: Numbers of children continuously insured during the YOL of interest or continuously insured until their death (n = 134) within this YOL are shown. *Children that didn’t survive perinatal hospitalization (if present) were excluded. (DOC 34 kb) [file 12887_2019_1424_MOESM1_ESM.doc]

**Supplementary Table 1**

**Number of children analysed by year of life:** Numbers of children continuously insured during the YOL of interest or continuously insured until their death (n=134) within this YOL are shown. *Children that didn’t survive perinatal hospitalization (if present) were excluded.

|  | **VLBW** | **LBW** | **Reference** | **Total** |
| --- | --- | --- | --- | --- |
| **1. YOL*** | 921 | 5,043 | 89,354 | 95,318 |
| **2. YOL** | 742 | 4,019 | 70,983 | 75,744 |
| **3. YOL** | 588 | 3,043 | 54,590 | 58,221 |
| **4. YOL** | 411 | 2,192 | 39,368 | 41,971 |
| **5. YOL** | 261 | 1,433 | 25,607 | 27,301 |
| **6. YOL** | 130 | 705 | 12,388 | 13,223 |
