# Supplementary material for: Impact of birthweight on health-care utilization during early childhood – a birth cohort study
Source: BMC Pediatr. 2019 Mar 1;19:69. doi: 10.1186/s12887-019-1424-8 (PMC6397462; doi:10.1186/s12887-019-1424-8)
Supplement: Supplementary file 2 — Perinatal Hospitalization. Gives information regarding the method of analysing perinatal hospitalization. (DOC 31 kb) [file 12887_2019_1424_MOESM2_ESM.doc]

**Perinatal hospitalization**

To analyse *frequencies of perinatal hospitalizations* all children insured from birth throughout the first seven days of life (DOL) or until death within the first seven DOL were included. About 1,897 infants had to be excluded due to the predefined eligibility criteria. Perinatal hospitalization was defined as first hospitalization starting within the first seven DOL. Healthy newborns are coded together with the mother’s delivery and thus data of these children do not contain a record of hospitalization. To analyse the *duration and costs of perinatal hospitalizations*, exclusively children with uncensored data of perinatal hospitalization were used. Censoring of data occurs due to change of health insurance during that stay or if hospitalizations last longer than the study period. Infants who died within the perinatal hospital stay are analysed separately with regard to duration and costs of perinatal hospital treatment. Out of 23,495 infants with perinatal hospitalization, data from 88 infants (0.37%) are censored.

We used χ^2^-test of independence to test the association between length of perinatal hospitalization and number of hospitalizations during the first year following perinatal hospitalization (both variables categorized) for each weight group.
