# Supplementary material for: Impact of birthweight on health-care utilization during early childhood – a birth cohort study
Source: BMC Pediatr. 2019 Mar 1;19:69. doi: 10.1186/s12887-019-1424-8 (PMC6397462; doi:10.1186/s12887-019-1424-8)
Supplement: Supplementary file 3 — Table S2. Perinatal hospitalization: Shown are number of infants insured during their first week of life (N) and that were perinatally hospitalized (N with periH, % with periH) stratified by year of birth and exposure group. (DOC 67 kb) [file 12887_2019_1424_MOESM3_ESM.doc]

**Supplementary Table 2**

**Perinatal hospitalization: Shown are number of infants insured during their first week of life (N) and that were perinatally hospitalized (N with periH, % with periH) stratified by year of birth and exposure group.**

| **Birth weight groups and subgroups** | | 2007 | | | 2008 | | | 2009 | | | 2010 | | | 2011 | | | 2012 | | | 2013 | | | Total | | |
| --- | --- | --- | --- | --- | --- | --- | --- | --- | --- | --- | --- | --- | --- | --- | --- | --- | --- | --- | --- | --- | --- | --- | --- | --- | --- |
|  |  | N | N with periH | %  with peri H | N | N with periH | % with periH | N | N with periH | % with periH | N | N with periH | % with periH | N | N with periH | % with periH | N | N with periH | % with periH | N | N with periH | % with periH | N | N  with periH | % with periH |
| **VLBW** | weight <1,000 g | 70 | 70 | 100.0 | 67 | 67 | 100.0 | 69 | 69 | 100.0 | 75 | 75 | 100.0 | 67 | 67 | 100.0 | 69 | 69 | 100.0 | 67 | 67 | 100.0 | 484 | 484 | 100.0 |
|  | weight 1,000-1,499 g | 98 | 98 | 100.0 | 105 | 105 | 100.0 | 110 | 110 | 100.0 | 119 | 119 | 100.0 | 95 | 95 | 100.0 | 104 | 104 | 100.0 | 114 | 114 | 100.0 | 745 | 745 | 100.0 |
|  | total* | 169 | 169 | 100.0 | 173 | 173 | 100.0 | 180 | 180 | 100.0 | 196 | 196 | 100.0 | 162 | 162 | 100.0 | 173 | 173 | 100.0 | 182 | 182 | 100.0 | 1,235 | 1,235 | 100.0 |
| **LBW** | weight 1,500-1,999g | 196 | 196 | 100.0 | 184 | 184 | 100.0 | 184 | 184 | 100.0 | 212 | 212 | 100.0 | 223 | 223 | 100.0 | 246 | 246 | 100.0 | 261 | 261 | 100.0 | 1,506 | 1,506 | 100.0 |
|  | weight 2,000-2,499g | 658 | 492 | 74.8 | 634 | 456 | 71.9 | 639 | 440 | 68.9 | 683 | 485 | 71.0 | 691 | 497 | 71.9 | 643 | 463 | 72.0 | 700 | 498 | 71.1 | 4,648 | 3,331 | 71.7 |
|  | total* | 857 | 690 | 80.5 | 828 | 649 | 78.4 | 830 | 630 | 75.9 | 903 | 702 | 77.7 | 926 | 731 | 78.9 | 896 | 712 | 79.5 | 974 | 769 | 79.0 | 6,214 | 4,883 | 78.6 |
| **Ref.** | weight ≥ 2,500g | 13,299 | 2,739 | 20.6 | 13,361 | 2,649 | 19.8 | 13,079 | 2,335 | 17.9 | 13,624 | 2,416 | 17.7 | 13,519 | 2,320 | 17.2 | 13,773 | 2,340 | 17.0 | 13,988 | 2,266 | 16.2 | 94,643 | 17,065 | 18.0 |
|  | no assignable weight | 1,776 | 39 | 2.2 | 1,834 | 49 | 2.7 | 1,822 | 41 | 2.3 | 1,976 | 46 | 2.3 | 2,032 | 45 | 2.2 | 2,305 | 48 | 2.1 | 2,432 | 44 | 1.8 | 14,177 | 312 | 2.2 |
|  | Total | 15,075 | 2,778 | 18.4 | 15,195 | 2,698 | 17.8 | 14,901 | 2,376 | 15.9 | 15,600 | 2,462 | 15.8 | 15,551 | 2,365 | 15.2 | 16,078 | 2,388 | 14.9 | 16,420 | 2,310 | 14.1 | 108,820 | 17,377 | 16.0 |
| **Total** | | 16,101 | 3,637 | 22.6 | 16,196 | 3,520 | 21.7 | 15,911 | 3,186 | 20 | 16,699 | 3,360 | 20.1 | 16,639 | 3,258 | 19.6 | 17,147 | 3,273 | 19.1 | 17,576 | 3,261 | 18.6 | **116,269** | 23,495 | 20.2 |
| *there are children with missing records of birth weight, but birth weight group (VLBW, LBW, refrence group) could be assigned by using P07-ICD-diagnoses which directly refer to a specific birth weight or by using admission weights of hospitalizations close to birth (for details, please refer to study protocol [Ref. 10] | | | | | | | | | | | | | | | | | | | | | | | | | |
