# Supplementary material for: Impact of birthweight on health-care utilization during early childhood – a birth cohort study
Source: BMC Pediatr. 2019 Mar 1;19:69. doi: 10.1186/s12887-019-1424-8 (PMC6397462; doi:10.1186/s12887-019-1424-8)
Supplement: Supplementary file 4 — Table S3. Death during perinatal hospitalization (in-hospital mortality): Shown are number of infants that died within perinatal hospitalization. For these children the health care cost for perinatal hospital treatment and the length of stay are represented as median with interquartile range (IQR). (DOC 36 kb) [file 12887_2019_1424_MOESM4_ESM.doc]

**Supplementary Table 3**

**Death during perinatal hospitalization (in-hospital mortality): Shown are number of infants that died within perinatal hospitalization. For these children the health care cost for perinatal hospital treatment and the length of stay are represented as median with interquartile range (IQR).**

|  | **VLBW** | **LBW** | **Reference** | **Total** |
| --- | --- | --- | --- | --- |
| **Infants with perinatal hospitalization** [N] | 1,205 | 4,845 | 17,357 | 23,407 |
| **Death** [n (% of N)] | 118 [9.8] | 28[0.6] | 53[0.3] | 199[0.9] |
| **Death within first week**  [n (% of N)] | 73 [6.1] | 18 [0.4] | 27[0.2] | 118[0.5] |
| **Days of hospitalization**  [Median (IQR)] | 3 (0-14) | 1 (1-12.5) | 7 (0-31) | 3 (0-17) |
| **Health care cost in €**  [Median (IQR)] | 4,625  (2,137-19,413) | 2,603  (2,223-28,124) | 8,668  (2,223-41,919) | 6,844  (2,162-25,744) |
