# Supplementary material for: Impact of birthweight on health-care utilization during early childhood – a birth cohort study
Source: BMC Pediatr. 2019 Mar 1;19:69. doi: 10.1186/s12887-019-1424-8 (PMC6397462; doi:10.1186/s12887-019-1424-8)
Supplement: Supplementary file 5 — Figure S1. A and B Length and costs of perinatal hospitalization and by birthweight: Shown are Boxplots of the length and costs of perinatal hospitalization. Children with missing record were excluded. (DOC 37 kb) [file 12887_2019_1424_MOESM5_ESM.doc]

**Supplementary figure 1A/1B**

**Length and costs of perinatal hospitalization and by birth weight:** Shown are Boxplots of the length and costs of perinatal hospitalization. Children with missing record were excluded.
