# Supplementary material for: Impact of birthweight on health-care utilization during early childhood – a birth cohort study
Source: BMC Pediatr. 2019 Mar 1;19:69. doi: 10.1186/s12887-019-1424-8 (PMC6397462; doi:10.1186/s12887-019-1424-8)
Supplement: Supplementary file 6 — Figure S2. Boxplots of the costs (in thousand Euro) of perinatal hospitalization by year of birth and birthweight: Children with missing record of birthweight were excluded. Outside values (observations below 1.Quartile − 1.5 IQR or above 3.Quartile + 1.5 IQR) are not shown in the graph. Simple unadjusted linear regression was calculated for these displayed costs and regression coefficients with 95% CI are reported. Note different scales for costs. (DOC 47 kb) [file 12887_2019_1424_MOESM6_ESM.doc]

**Supplementary figure 2**

**Boxplots of the costs (in thousand Euro) of perinatal hospitalization by year of birth and birth weight:** Children with missing record of birth weight were excluded. Outside values (observations below 1.Quartile − 1.5 IQR or above 3.Quartile + 1.5 IQR) are not shown in the graph. Simple unadjusted linear regression was calculated for these displayed costs and regression coefficients with 95% CI are reported. Note different scales for costs.
