# Supplementary figures and images for: Impact of birthweight on health-care utilization during early childhood – a birth cohort study
Source: BMC Pediatr. 2019 Mar 1;19:69. doi: 10.1186/s12887-019-1424-8 (PMC6397462; doi:10.1186/s12887-019-1424-8)

Relative frequency of children  
with respective number of hospital stays

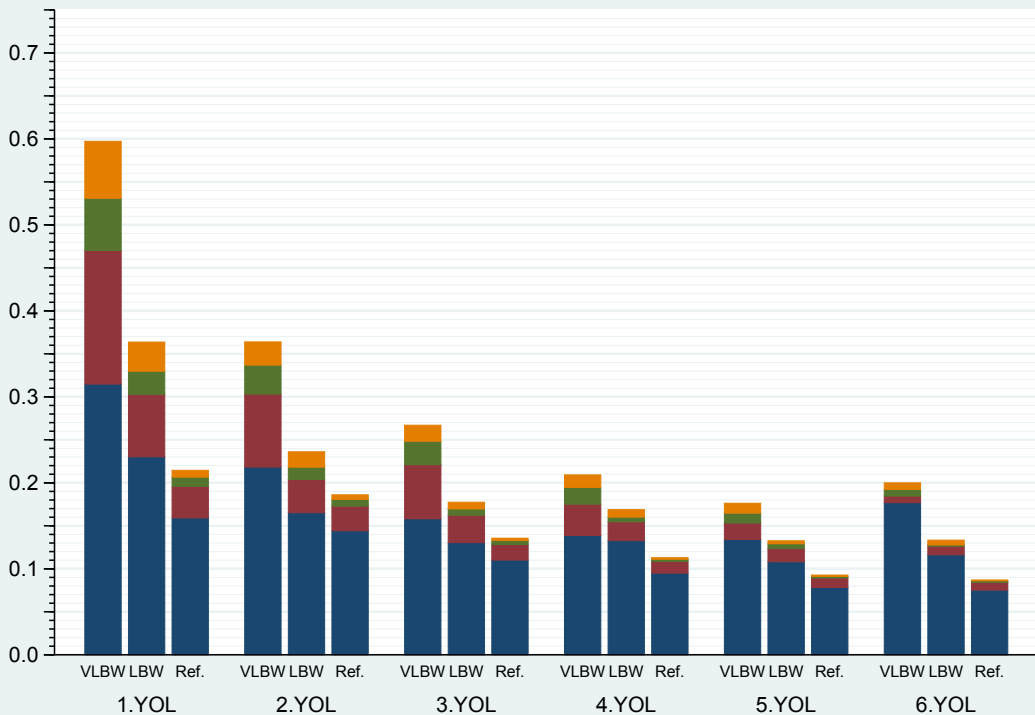

Supplement: Supplementary file 7 — Figure S3. Number of hospital stays excluding perinatal hospitalization and observational admissions by YOL and birthweight: Shown are the relative percentages of VLBW-, LBW- and reference-infants with 1(blue), 2(red), 3(green) or more than 3(orange) hospitalizations in the respective year of life (YOL) excluding perinatal hospitalization and excluding all hospitalizations just for the reason of vaccination or further circumstances not encoded as disease (Z-codes of ICD-10-GM). (DOC 34 kb) (PDF 114 kb) [file 12887_2019_1424_MOESM7_ESM.pdf]
