# Supplementary material for: Impact of birthweight on health-care utilization during early childhood – a birth cohort study
Source: BMC Pediatr. 2019 Mar 1;19:69. doi: 10.1186/s12887-019-1424-8 (PMC6397462; doi:10.1186/s12887-019-1424-8)
Supplement: Supplementary file 8 — Table S4. Characteristics of the study population: Shown are the total numbers of children born alive in Saxony stratified by year of birth, sex and birthweight given by the Federal Statistical Office of Germany and the Statistical Office of the Free State of Saxony. The same numbers are given for the study population. (DOC 37 kb) [file 12887_2019_1424_MOESM8_ESM.doc]

**Supplementary Table 4**

**Characteristics of the study population:** Shown are the total numbers of children born alive in Saxony stratified by year of birth, sex and birthweight given by the Federal Statistical Office of Germany (<https://www-genesis.destatis.de/genesis/online/>) and the Statistical Office of the Free State of Saxony (https://www.statistik.sachsen.de/GBE/Gesundheit_Start.htm). The same numbers are given for the study population.

| **Year of birth** | **Children born alive in Saxony** | | | | **Children born alive and AOK PLUS-insured at birth** | | | |
| --- | --- | --- | --- | --- | --- | --- | --- | --- |
|  | **Total** | **female (%)** | **birthweight below 1500g (%)** | **birthweight 1500-2500g (%)** | **Total** | **female (%)** | **birthweight below 1500g (%)** | **birthweight 1500-2500g (%)** |
| 2007 | 33,858 | 16,434 (48.5) | 319 (0.9) | 1,725 (5.1) | 16,308 | 7,799 (47.8) | 172 (1.1) | 869 (5.3) |
| 2008 | 34,411 | 16,753 (48.7) | 323 (0.9) | 1,695 (4.9) | 16,443 | 8,059 (49.0) | 176 (1.1) | 847 (5.2) |
| 2009 | 34,093 | 16,486 (48.4) | 381 (1.1) | 1,747 (5.1) | 16,147 | 7,684 (47.6) | 183 (1.1) | 845 (5.2) |
| 2010 | 35,091 | 17,171 (48.9) | 372 (1.1) | 1,851 (5.3) | 16,932 | 8,357 (49.4) | 200 (1.2) | 927 (5.5) |
| 2011 | 34,423 | 16,735 (48.6) | 330 (1.0) | 1,872 (5.4) | 16,855 | 8,178 (48.5) | 165 (1.0) | 937 (5.6) |
| 2012 | 34,686 | 16,936 (48.8) | 355 (1.0) | 1,859 (5.4) | 17,382 | 8,428 (48.5) | 179 (1.0) | 912 (5.2) |
| 2013 | 34,800 | 16,757 (48.2) | 336 (1.0) | 1,831 (5.3) | 18,099 | 8,728 (48.2) | 190 (1.0) | 1,004 (5.5) |
| Total | 241,362 | 117,272 (48.6) | 2,416 (1.0) | 12,580 (5.2) | 118,166 | 57,233 (48.4) | 1,265 (1.1) | 6,341 (5.4) |
